# Practical Remarks upon the Treatment of Simple and Complex Forms of Intermittent Fevers

**Published:** 1849

**Authors:** A. H. Howland

**Affiliations:** President of the Association


THE NORTH-WESTERN

### MEDICAL AND SURGICAL JOURNAL.

Vol. II.] APRIL AND MAY, 1849. [No. 1.

### Part I.—Original Communications.

### ARTICLE I.

*Practical Remarks upon the Treatment of Simple and Complex Forms of Intermittent Fevers.* Read before the Ottawa Medico-Chirurgical Association. By A. H. Howland, M.D., President of the Association.

I trust that, from a practice of twenty-five years exclusively in districts of country rife with all the protean forms of disease that emanate from what is termed "*Malaria*," my remarks may be of some value in a practical point of view, at least to the junior members of the profession. I have quite as little desire as ability to make a display of professional erudition by an attempt to present you with a learned discourse upon medical science, and have therefore supposed that to give you a synopsis of my experience in the treatment of the intermittent and remittent fevers and some of their varied complications, as they prevail in this state, would be far more acceptable if not more beneficial to you than anything else I could offer.

All the theoretical knowledge which the learned Professors in our Eastern Medical Institutions can impart to the student of medicine, will never qualify him to treat with success the endemic fevers of this climate, especially when they similate and are complicated with other diseases; or to treat them in a manner that will satisfy the conscience of one who has a correct view of the value of human life, and the great responsibilities of medical men, unless these professors have had an *extensive practical experience themselves*, in such diseases. Nothing but clinical observation and experience will enable us to detect the true pathological condition of the system in the complicated forms of disease that appear in malarious districts; and if we fail in this, our prescriptions may be useful, or they may prove injurious, according to circumstances.

When I first came here, fourteen years ago last October, and for several years after, the malarious influence was so strong that no other disease was permitted even a temporary abode in our mortal frames. Thus it was in Western New York, when I first settled there in 1823. But as this influence gives way by the improvement of the country and the acclimation of the inhabitants, the thousand nameless ills that "flesh is heir to," begin to assert their prerogative to reign in the flesh, and if they cannot reign alone are content to share a divided empire.

Most of you, gentlemen, are yet young in the profession, and in common with all mankind, I have no doubt, feel full confidence in yourselves to treat all diseases scientifically, and with approved skill. At least so thought I when I commenced the practice; and in this opinion, perhaps, we do not greatly err. But the error lies in our inability, without experience, to tell what the disease really is that we are called to treat; or, in other words, to discover the true pathological condition of the system. Here is the great obstacle that lies in the way of successful practice. This removed, a well grounded theoretical knowledge, without a day's experience, is all that is essential to correct and appropriate treatment. Therefore, to be able to discern this condition of the system is of the most momentous consequence, not only to the patients but also to the success and reputation of the physician. I cannot better illustrate the truth of this position than to say that I lost more patients in proportion to the number treated, other things being equal, during the first five years of my practice than I have during the last fifteen. And if you will keep a record of the cases you treat and their results, and continue the practice as long as I have, you will find it not only useful but confirmatory of my own experience. You will *give less medicine and lose fewer patients* under a more enlarged experience.

Happily for the human family, it is no longer deemed necessary, by the majority of our profession at least, to salivate, or to scrape the stomach and intestines with drastic purgatives and emetics, in order to cure the autumnal remittent and intermittent fevers. To this great change I attribute some portion of the decrease of deaths in my own practice. For many years, in the treatment of *all* diseases, I have avoided ptyalism by every precaution compatible with a judicious use of calomel. I seldom give emetics—never in the uncomplicated forms of these fevers in the sultry season, or even in September—especially mineral emetics. It may seem paradoxical to say that what is correct and successful practice in these fevers *one year*, may be improper and even fatal the next, yet such has been my experience. This is occasioned by strong local determinations to this or that important organ, to the presence or absence of epidemic influence and its character when present. As a familiar example, the epidemic influence of the black tongue, (or the epidemic erysipelas as it was called,) although but a few sporadic cases occurred here, rendered bleeding almost certainly fatal in the treatment of these fevers, unless it was promptly followed by powerful tonics and stimulants. If the fevers present determinations to the brain and serous membranes, depletion is far more admissible than when the determination is to the mucous tissues, or to the parenchymatous substance of any important organ. Some years emetics are borne with safety, while in others they are positively fatal. My practice in the treatment of these fevers has been, for years, very plain and simple, and I know far more successful than formerly, when I used a much greater variety of medicines than I now do, and dealt most of them out with greater liberality. I believe that every case of these fevers, as they occur here in ordinary constitutions, when uncomplicated, is curable. Sedatives, mild purgatives and tonics, or, in other words, morphine, quinine, calomel, rhubarb, and sup. carb. of soda constitute not only my main reliances, but they are about all the articles I either purchase or carry with me from June to November. During the remainder of the year, I add to this list tartar emetic and ipecacuanha. Some years I find many cases that require bleeding, in others but a few. I never bleed during the decline of a paroxysm, unless it be attended with local inflammation. I am generally called on the approach of the second paroxysm of these fevers. The treatment I have found most successful is the following:

If the case requires bleeding to bring the system into a condition favorable for the influence of quinine, bleed, and prescribe calomel, 15 grs., morphine ¼ gr., to be mixed and taken at once, and cold water ad libitum. I leave at the same time calomel 5 grs., morphine ¼ gr. to be taken in from four to six hours. I also leave, on my first visit, quinine 20 grs., rhubarb 20 grs., mixed and divided into three portions. Into one of these I put morphine ¼ gr. and direct this one to be taken at that hour of the night or morning when it is probable the fever will be approaching or at its greatest remission or intermission as the case may be, following it with the other two portions at intervals of one or two hours, as the intermission is expected to be longer or shorter, but to be given though there be no remission. I make it a point to visit my patients next day between ten A. M. and two P. M. If the bowels have not moved, I order rhubarb and soda 10 grs. of each, to be mixed and taken in a spoonful or two of cold water, and repeat the same every two hours till it operates, accompanied with cold acidulated drinks if required by the patient. I then leave quinine, 15 grs. divided into three portions with ¼ gr. morphine in one of them, to be taken first in the latter part of the night or early part of the morning, and followed by the other two as before. In ordinary cases, this treatment, followed by 5 grs. quinine every morning for a few mornings, and keeping the bowels regular with rhubarb and soda, is all that is necessary to effect a cure. Blisters and the old fashioned fever powders made of camphor, ipecac., opium, and nitre, I have long since abandoned as positively injurious, for in many of the cases the stomach is in a congested or phlogosed state, rendering all irritating or stimulants highly improper. Yet, to my surprise, I frequently see them prescribed, and that by new fledged M. D.s fresh from distinguished medical schools. If the quinine be good nearly every case of plain, uncomplicated fever, as it occurs here, will yield to the above treatment in forty-eight hours. Of late, owing to the diminished power of the quinine, I have been obliged to nearly double the dose. A judicious use of these remedies by increasing or decreasing their quantities and varying their proportions, together with local bleeding to meet the varied circumstances of age, sex, and pathological condition in different individuals, and in different seasons, has proved, in my hands, for many years past, preeminently successful.

The complicated form of this disease which destroys the greatest number of lives in this state is that which prevailed here last spring and this winter — generally called winter fever, or lung fever, by the people and by many physicians. The seat of inflammatory action, during the incipient stages, is most frequently the bronchia, and if permitted to run its course, it finally involves the lungs and not unfrequently the liver—constituting a well marked case of pneumonia biliosa. This complex disease has destroyed more lives for three years past in this and adjoining states, so far as my observation extends, than all other causes of death combined. This fatality has attended it, not, as I apprehend, because it is incurable, but becase the depressing influence of the malarious poison, latent in the system, is overlooked or not recognized by the physicians, and not even suspected by those who have lately arrived from the eastern states where these fevers do not prevail. This latter class of physicians treat it as they would a case of pneumonia in New England, which only hurries the sufferers to the grave.

I will give you the results of my experience in the treatment of this disease. I have found it eminently successful, so much so that during the first six months of last year I lost but two patients within the limits of this corporation, who came under my care before they had been prescribed for by others. If the patient be a stranger to me, my first inquiry is—where have you lived during one or two years last past? If his reply satisfies me that he has not been exposed to the malarious influence during the two previous years, I treat the case as I would one presenting the same or a similar train of symptoms in New England. If, on the contrary, he has been exposed to such an influence, still, as a general rule, I bleed either generally or locally, or both. But whether I bleed or not emetics, calomel, and quinine are my sheet anchor of hope. If the attack be recent, it will be easy to detect a periodicity in the disease, marking the footsteps of the insidious foe, "malaria;" but if it has been running its course for three, four, or five days, then the inflammatory action is by far the most, if not the only ostensible feature of the disease rendering the febrile movement continuous. Be this as it may, as a general rule, if the patient has resided in a district of country during any considerable portion of the last twelve months, where these fevers are known to prevail, I adopt the same course of treatment that I would if I knew him to be laboring under the depressing influence of malaria. I bleed as above stated, and prescribe an emetic of tartrate of antimony in sufficient quantity to operate when combined with one-eighth grain of morphine. When so combined, I

know from experience in my own person, that it is much less distressing and irritating to the stomach, especially if mucilages are given during its operation. If business permits, I make a point to remain and see that the liver is acted on and healthy bile thrown up. After which I prescribe from five to fifteen grains of calomel, with one-fourth grain morphine and one-fourth grain tartar emetic, or five grains ipecac., to be taken about three or four hours after the operation of the emetic; and I repeat this powder with a diminished quantity of morphine every three or four hours, according to the violence of the inflammatory action, till from two to four powders are taken. I endeavor so to time it that the foregoing prescriptions are gone through with by two o'clock A. M. I also leave three powders containing each five grains quinine, one-eighth grain morphine, one-eighth grain tartar emetic, and five grains rhubarb, directing them to be taken at four six, and eight o'clock, A. M. I leave also directions that if these medicines do not move the bowels freely by ten or eleven o'clock, A. M., to have them moved by injections or castor oil, or rhubarb and soda. I endeavor to return at about two P. M., next day, and then repeat the bleeding, general or local, or both, or neither, according to the violence of the inflammation, and if the disease does not appear to be yielding rapidly, I repeat the emetic but not the tartar emetic alone, but combined with ipecac. or eupatorium. And if the liver appears to be involved in the inflammatory action, I repeat the calomel in diminished doses, and follow it again with quinine combined as before. and during the same portion of the twenty-four hours—moving off the bowels next day with oil or rhubarb and soda if necessary. To deplete and give tonics on the same day may appear strange to some of you, yet experience has taught me that such treatment is not only compatible with my views of the pathology of this disease, but also that it is eminently successful. The quinine would not be borne without increasing the inflammatory action, if unaided by depletion, and depletion unaided by the quinine to destroy the depressing influence of the malaria, would only hasten the patient to that "bourne whence no traveler returns." A repetition of local bleeding and of the quinine is frequently necessary for several days and in many cases of the emetic also; but if judiciously applied the disease is sure to yield.

In all the post mortem examinations I have witnessed in this disease, death has been the consequence of inflammatory action, yet it stole on insidiously, unattended with much pain or uneasiness until irreparable mischief was done—hence we see the importance of being upon our guard, and of treating its incipient stages with prompt and efficient remedies, and holding no truce nor parley with the skulking foe, until the natural and healthy secretions of the system are fully established.

In this disease I sometimes blister, *but never while general depletion is necessary*, nor hardly ever in children.

I have seen more masked, anomalous, and complex cases of intermittent and remittent fevers during the last eighteen months than I have in five years before — since 1841-2. There is hardly a disease that prevails in the United States, whose mantle the foul fiend has not assumed. The whole catalogue of nervous diseases and all the inflammatory affections have been imitated with a fidelity and a semblance of truth so strong, as in some instances to baffle the scrutiny of the most experienced in the profession. As an instance in point, allow me to relate a case. A year ago last December I was called in the night to see the Hon. Mr. M———, who was said to be dying at the Fox River House. On my arrival I found him bolstered up in bed laboring under what he called, and what his physician in Joliet called, and what in fact appeared to be, a violent fit of asthma; so violent indeed that suffocation seemed inevitable, and probably would have taken place in a recumbent position. The coughing was incessant, and the quantity of mucous slightly tinged with blood that he threw up was enormous. I learned from his wife—for it was with the greatest difficulty that the patient could speak at all—the attacks had occurred irregularly at intervals varying from one to three or four weeks, for three or four months past; that his physicians had told him it was asthma, and treated it accordingly; and that he had, that morning, left Joliet for St. Louis, in usual health, but with an ample stock of his medicine for the asthma in case of an attack. I pronounced it a masked intermittent and proposed bleeding. To this he objected, saying he knew it to be asthma. I soon reasoned him into compliance, and drew from his arm twenty ounces of blood and administered an emetic. The bleeding produced immediate but only partial relief—the emetic was combined with morphine, and, as soon as it operated, gave him entire ease. I then gave eight grains quinine combined with morphine and rhubarb, and followed it with five grains more in two hours. I also furnished him with fifty grains quinine, made into powders of five grains each, with morphine and rhubarb, three of which I directed him to take during the day, and after that one every morning until all were taken. In the morning, at ten o'clock, sixteen hours from the commencement of the attack, I sent him on his way to St. Louis with instructions to have his asthma treated as an ague, since which he is no longer troubled; finding a prompt remedy in quinine and emetics, without the aid of his asthmatic physician.

I scarcely meet a case of disease in any one who has re-resided in the Western States during one summer, that does not require quinine. But to give it uncombined with morphine, ipecac., tartar emetic, or purgatives, one or all, as the case may seem to require, or without bleeding, would destroy instead of cure in all cases attended with any considerable amount of inflammatory action. But judiciously administered in conjunction with these, it is all powerful to cure. I ask for no more potent remedy except in a few cases similating the nervous affections, as tic doloreaux or sciatica. In a few of these I have found it to fail, and have had recourse to arsenic which has succeded after quinine, in conjunction with the most approved preparations of iron, had entirely failed. When giving quinine in large and repeated doses in some bad cases during the last spring and winter without the desired result, I have sometimes paused in doubt as to the propriety of the treatment I was pursuing, yet, on a careful revision of the case, I have invariably had to return to its use ; but sometimes not till after prescribing another emetic or bleeding, and then have found it successful. It is now so adulterated and so much of it is made from other barks that no certain reliance can be placed on a given number of grains. It must be given *till the desired effect is produced.* Strychnine I have never used, and regret that it has been introduced as a remedy, believing, as I do, that more damage than good will be the result. I know of six deaths within the last two months from its use, and have no doubt that hundreds will fall a sacrifice to its influence, for it is scarcely a safe remedy in the hands of the most skilful to say nothing of the unskilful, the charlatans, and some of the common people, who are already beginning to use it. They doubtless think that they can prescribe it as well as quinine or morphine—as one poor man did a few weeks since at Homer, in this county, who gave it to five of his children, four of whom died in horrible agony in thirty minutes.

With these remarks I close, not, however, without expressing the hope, that you, gentlemen, will feel sufficiently interested in the progress of medical science, and the importance of associating for our mutual benefit, to sustain this society, to hold regular meetings, and to appoint delegates to the national convention. Our numbers will fast increase with the growth and prosperity of the town and country, and much may be done, by proper association to maintain the dignity of the profession, command the respect of the community, suppress qackery, and advance our mutual interests.

